# New Appliances and Things Medical

**Published:** 1898-06-25

**Authors:** 


### NEW APPLIANCES AND THINGS MEDICAL.

[We shall be glad to receive, at our Office, 28 & 29, Southampton Street, Strand, London, W.C., from the manufacturers, specimens of all new preparations and appliances which may be brought out from time to time.]

### LIEBIG'S EXTRACT OF MEAT AND MALT WINE.—CORONA BRAND.

(Dr. Brainz, New Hirst Mill, Bingley, Yorks.)

This new preparation is a mixture of extract of malt, extract of meat (Liebig), and port wine. It is, therefore, a food, a stimulant, and a digestive in one. It is pleasant to the palate, and its invigorating effects can be almost immediately realised after swallowing a small portion.

### NEW ENEMA.

(S. Maw, Son, and Thompson, 7-12, Aldersgate Street, London, E.C.)

The new enema which has recently been brought out by this firm possesses two special advantages which should appeal to those who use these surgical accessories—firstly, it is entirely of British manufacture; secondly, it is of very low price, *i.e.*, 21s. per dozen. It is made of red rubber, and from our experience of it it has a good life. The quality of the rubber appears vastly superior to that of the cheap German-made variety, with which, however, it stands at little disadvantage as regards price owing to the enterprise of the manufacturers and the large number which is being daily turned out.

### COCA KOLA WINE.

(L. Poirson, Birkdale, Southport.)

We have received two samples of the above wine, one "dry," and the other "rich." Both appear to be good invalid tonics, containing the active principles of coca and kola. Whether the "dry" or the "rich" brand should be chosen, depends on the constitutional condition of the patient. There can be no doubt that such a wine as that prepared by L. Poirson, when used with discretion in certain cases of nervous exhaustion, is of the utmost value. When consumed indiscriminately, without medical supervision, its invigorating properties may, however, be a danger rather than a benefit.

### KRYOFINE: A NEW ANTI-PYRETIC.

(D. Misell, 65, Basinghall Street, London, E.C.)

Kryofine is, like phenacetin, a p-phenetidin derivative, and, like it, has powerful anti-pyretic properties. It forms white, odourless crystals, which have no taste. Its solubility in water is 1·52 in boiling water and 1·600 in cold water. As an anti-neuralgic kryofine may also have valuable properties; but, doubtless, its anti-pyretic powers are those on which it will have to rely to find a permanent place in the physician's practical pharmacopœia. In dosage it should be administered in quantities of from 5 to 10 grains.

### TURTLE SOUP.

(Skinner's Queensland Delicacies. Importer, A. Chadwick, 7, Carlton Street, Regent Street, London, S.W.)

We have made trial of several varieties of turtle soup supplied in tins by this firm ready for use. The invalid turtle is what we have paid most attention to, as it is most likely to prove of use to the majority of our readers. It appears to be a most carefully-prepared delicacy of the highest quality, and, from its excellent nutritious qualities, to be suitable to invalids who are recovering from serious illness and require something that is at once nutritious, digestible, and tempting to the appetite.

